# Dare to take care of yourself: the relationship between psychological courage and the intention to undergo mammography screening

**DOI:** 10.3389/fpubh.2026.1778940

**Published:** 2026-06-09

**Authors:** Grzegorz Pajestka, Katarzyna Skałacka

**Affiliations:** Institute of Psychology, Opole University, Opole, Poland

**Keywords:** anxiety, mammography, preventive behavior, psychological courage, screening test

## Abstract

**Purpose:**

This study aimed to investigate the role of psychological courage (PC)—understood as the willingness to act despite fear or anxiety—in predicting women's intentions to undergo mammography screening. While PC has been linked to adaptive behavior in challenging contexts, its relevance in preventive healthcare remains largely unexplored.

**Methods:**

A sample of 292 women aged 45–74 with no history of breast cancer completed measures assessing PC, anxiety, cancer worry, perceived risk, and past screening behavior. Hierarchical regression analysis was used to determine whether PC predicted screening intentions above and beyond traditional psychological predictors.

**Results:**

PC emerged as a significant predictor of the intention to undergo mammography, even after accounting for anxiety, cancer worry, perceived risk, and prior screening. No evidence was found for interaction effects between PC and negative emotional variables.

**Conclusions:**

These findings challenge fear-based models of health behavior by highlighting the role of positive psychological traits—specifically courage—in motivating preventive actions. As a potentially modifiable trait, PC may serve as a novel target for health communication and intervention strategies aimed at increasing participation in cancer screening programs.

## Introduction

### Psychological courage

Psychological courage (PC) is a relatively recent concept in psychology, originally introduced by Putman (1) to describe the act of facing irrational fears and personal struggles in emotionally challenging situations. Although initially discussed in theoretical contexts (e.g., (2)), PC shares key features with Rachman's (3) definition of courage as persistence despite fear—a view later operationalized by Norton and Weiss (4) in the Courage Measure. Given this overlap, Pajestka (5) adopted the term PC to capture the essence of acting in spite of fear or anxiety, as measured by this scale (see also (6)). This framing distinguishes PC from moral or physical courage, which typically involve prosocial action and do not require the experience of fear (7, 8). In contrast, PC is centered on intrapersonal struggle, with fear as a defining element. PC, as measured by the Courage Measure or its short form (9), has been linked to actual behavior—such as longer speech duration in individuals with public speaking fear (10) and approach behavior toward feared stimuli (4, 5, 11). It also predicts outcomes beyond fear, anxiety, or distress, suggesting unique explanatory value. Within the Reinforcement Sensitivity Theory (RST; (12)), PC is thought to buffer the negative effects associated with behavioral inhibition system (BIS), supporting its role as a resource for managing negative emotions (13). However, its potential role in shaping how negative emotions relate to health behavior—such as screening—remains unexplored.

Although PC shares conceptual space with constructs such as self-efficacy, resilience, and approach motivation, it is theoretically distinct. Approach motivation reflects a general tendency to pursue positive outcomes, whereas self-efficacy refers to beliefs about one's capability to successfully perform actions. In contrast, PC involves action undertaken despite fear, uncertainty, or anticipated negative consequences. Similarly, while resilience concerns adaptation to unavoidable stressors, PC is particularly relevant in situations that can be avoided but are nonetheless confronted despite fear. Therefore, PC may be particularly relevant in screening contexts, enhancing attendance despite fear, even in the absence of strong efficacy beliefs or adaptive emotional regulation skills.

### Breast cancer

Breast cancer is the most common cancer among women globally, with rising incidence, particularly in those over 50 (14). Current estimates suggest that one in 20 women will be diagnosed and one in 70 will die from the disease. If trends continue, global incidence and mortality may rise by 38 and 68% by 2050. Serial mammography remains the most effective method for early detection and mortality reduction (15, 16).

Breast cancer rates and screening policies vary by country. Higher-HDI nations tend to have lower mortality, reflecting more effective healthcare systems (14). For example, up to one-third of breast cancer deaths in sub-Saharan Africa could be prevented through screening (17). Despite national programs, countries like China and Poland show low screening rates (18, 19), highlighting the need to understand barriers to participation.

## Cancer worry

Emotions such as anxiety and fear play a central role in decisions about cancer screening, acting as both motivators and barriers. Worry is typically defined as repetitive, future-focused negative thinking related to uncertainty (20, 21). Unlike perceived risk, which is purely cognitive, or anxiety, which is broader and physiological, worry involves targeted emotional responses. While pathological worry occurs in anxiety and depressive disorders (22), adaptive worry can facilitate preventive behaviors like screening.

In cancer prevention, worry is commonly treated as a negative emotional reaction to cancer threat (23), and is often operationalized as one form of broader cancer fear (24). Cancer fear has been linked to both increased and decreased screening attendance, depending on the aspect of fear examined. For example the procedure itself—related to pain or embarrassment—can deter participation (25, 26). Generalized cancer fear tends to promote screening, while more specific fears often reduce it (27). A recent meta-analysis found varied links between anticipatory anxiety and breast cancer screening, including positive, negative, and null effects (28), with moderators such as perceived risk or family history influencing outcomes. The most recent large-scale study confirmed a positive association between breast cancer worry and screening intentions (29).

In sum, fear related to cancer can play a dual role in screening behavior. While cancer worry may motivate screening, cancer fear has also been associated with lower screening uptake, suggesting that it may function as a barrier in some cases (for review see (30)).

## Current study aims

Emotions play a critical role in health-related decision-making (31), and understanding their impact on cancer screening behaviors may be important for improving early detection and outcomes. This study examines the relationships between psychological courage (PC), breast cancer worry (BCW), and mammography screening intentions. Previous research has shown PC to be negatively associated with negative emotions and positively linked to behavioral outcomes and wellbeing (6, 11). PC has also been shown to moderate the impact of negative emotions on behavior (5), suggesting it may positively relate to screening intentions. However, given its buffering potential, PC may also attenuate the motivating role of cancer-related worry. In situations where worry would otherwise promote screening intentions, higher levels of PC may weaken this association by reducing the extent to which worry translates into action. Although both beneficial and detrimental effects of PC are plausible, the former aligns more with existing evidence and PC's conceptualization as a personal resource (1). To account for established predictors of screening behavior, we included common covariates: age, education, prior screening, personal/family cancer history, perceived risk, and trait anxiety.

In summary, this study aimed to test whether PC predicts mammography screening intentions beyond BCW, trait anxiety, and other controls. Additionally, we examined whether PC moderates the relationships between BCW, trait anxiety, and screening intentions. To date, no research has explored PC in relation to preventive behavior or the positive effects of negative emotions, making this study novel within and beyond the context of breast cancer prevention. Ethical approval was granted from the institutional review board at the authors' university (no. 82/2024). Data files and codes for this paper are provided in the Open Science Framework (OSF) at https://osf.io/vd2p9/?view_only=a0cdcf560f954a498e6df0758e39d348.

## Method

### Participants

Sample size for hierarchical regression was estimated using effect sizes from prior studies (4, 5). With PC effects of *f*^2^ = 0.136 and PC × anxiety interaction *f*^2^ = 0.105, and assuming α =0.05 and power = 0.80, at least 125 participants were needed to detect a significant R^2^ change with six predictors; 172 participants were required when adding two interaction terms (32).

The initial sample included 306 Polish women aged 45–74 (*M* = 53.51, *SD* = 7.44), recruited via Facebook. Social media recruitment was used to efficiently reach screening-eligible women. The only inclusion criterion was reaching the minimum age for mammography eligibility under the national breast cancer screening program, which is 45 years in Poland. Fourteen women who reported a current or past breast cancer diagnosis were excluded. Their motivational and emotional responses to screening may differ significantly from those of women without a cancer history, and their group sizes were too small to analyze separately. The final sample included 292 women (*M* = 53.33, *SD* = 7.43). Sample characteristics are presented in Supplementary Table S1.

### Measures

Psychological Courage (PC). PC was assessed using the 6-item Courage Measure (9), based on the definition of courage as persistence despite fear (4). Items (e.g., “I tend to face my fears”) were rated on a 7-point Likert scale (1 = strongly disagree to 7 = strongly agree). The scale has been previously used in research on PC (6, 13), supporting its applicability in this context. Internal consistency was high (α = 0.88 in the present study; α = 0.84 previously). Additional details on the measure are provided in the Supplementary materials.

Breast Cancer Worry. The 4-item Breast Cancer Worry Scale (33) was used to measure worry about developing breast cancer. Various versions of the scale exist, but we used the original 4-item version and standardized responses to a 5-point Likert scale (1 = not at all to 5 = very much). One item was rephrased for consistency: “What is your current level of anxiety about the results of future mammograms?” was changed to “How anxious are you about the results of future mammograms?” Other items included: “How much do you currently worry about getting breast cancer someday?”, “How much do worries about breast cancer impact your mood?”, and “How much do worries about breast cancer impact your daily activities?” Internal consistency was high (α = 0.86).

Mammography Intention. Intentions were assessed with three items based on cancer prevention literature (e.g., (34, 35)). Items included: (1) “Are you planning to undergo a mammogram?”, (2) “Do you plan to have regular mammograms (e.g., every 2 years)?”, and (3) “Are you planning to get a mammogram soon (for example, within the next year or, if you did so recently, within the next 2 years)?” Responses were rated on a 5-point Likert scale (1 = definitely no to 5 = definitely yes). To improve reliability over single-item measures, a composite index was used, showing excellent internal consistency (α = 0.90).

Perceived Risk. Perceived breast cancer risk was measured with two items adapted from McGinty et al. (36), using a 5-point scale (1 = extremely unlikely to 5 = extremely likely). Items included: “How likely do you think you are to have breast cancer?” and “How likely do you think you are to develop breast cancer in your lifetime compared to other women your age?” Reliability was acceptable (α = 0.79; Spearman-Brown = 0.79).

Trait Anxiety. Trait anxiety was assessed with the 10-item BIS-Anxiety subscale from the IPIP-BIS/BAS (42). Participants rated how well each statement described them on a 5-point scale (1 = very inaccurate to 5 = very accurate). Example items included “Often worry about things that turn out to be unimportant” and “Become overwhelmed by events.” Internal consistency was high (α = 0.90 in this study; α = 0.84 previously).

### Analysis

Data were analyzed using IBM SPSS Statistics 29. Descriptive statistics and bivariate correlations were followed by hierarchical regression analyses. Assumptions were checked, and all continuous predictors were z-scored to reduce multicollinearity due to interaction terms. In Step 1, breast cancer worry (BCW), trait anxiety, perceived risk, and control variables were entered; Step 2 added psychological courage (PC); Step 3 included interaction terms (PC × BCW, PC × trait anxiety). Categorical variables (family history, previous mammography, education) were dummy-coded.

## Results

### Preliminary analyses

Descriptive statistics (Supplementary Table S2) showed that skewness and kurtosis values for predictors were within acceptable limits (< |1|), suggesting approximate normality. The dependent variable showed negative skewness and a high mean, indicating that most women had positive intentions for mammography. Table 1 shows that these intentions were correlated with psychological courage (PC) and perceived risk, but not with breast cancer worry (BCW) or trait anxiety.

**Table 1:** Zero-order correlations between focal study variables.

Variable	Intention	PC	BCW	perceived risk
Intention	—			
PC	0.21^***^	—		
BCW	0.06	−0.12^*^	—	
Perceived risk	0.13^*^	−0.05	0.34^***^	—
Trait anxiety	0.04	−0.29^***^	0.41^***^	0.13^*^

Intention, intention to undergo mammography; PC, psychological courage; BCW, breast cancer worry.

^*^P < 0.05, ^***^P < 0.001.

### Multiple hierarchical regression analyses

Before hierarchical regression, assumptions were checked (37). P–P plots (probability–probability plots) and scatterplots showed no violations of normality, linearity, or homoscedasticity. Multicollinearity was low, with variance inflation factors (VIFs) below 1.5.

As shown in Table 2, breast cancer worry (BCW) was not a significant predictor of mammography intention, whereas trait anxiety (step 3), perceived risk, age, and prior mammography were significant predictors. None of the planned interaction terms (PC × BCW, PC × trait anxiety) were significant.

**Table 2:** Results of hierarchical regression analysis for variables predicting intention to undergo mammography.

Predictor	Step 1	Step 2	Step 3
BCW	0.007	0.004	0.002
Trait anxiety	0.040	0.114	0.122^*^
Perceived risk	0.117^*^	0.116^*^	0.123^*^
Age	−0.174^**^	−0.143^*^	−0.139^*^
Education	0.16	−0.018	−0.023
Family history	0.054	0.057	0.048
Previous mammography	0.428^***^	0.419^***^	0.417^***^
PC		0.250^***^	0.249^***^
PC × BCW			0.045
PC × trait anxiety			−0.117
*R* ^2^	0.162	0.217	0.227
Δ*R*^2^	0.162	0.055	0.010
Sig. Δ	0.001	0.001	0.174

BCW, breast cancer worry; PC, psychological courage.

Standardized regression coefficients (β) are reported.

^*^P < 0.05, ^**^P < 0.01, ^***^P < 0.001.

Additionally, we tested whether it moderates the relationship between PC and intention by including an interaction term (PC × prior mammography). This interaction was also not significant (β = −0.063, *P* =0.543).

## Discussion

This study explored the link between psychological courage (PC) and intention to undergo mammography screening in 292 women without a breast cancer history. PC was hypothesized to predict intention beyond known factors like worry, anxiety, perceived risk, family history, prior screening, age, and education. Results confirmed this: PC accounted for additional variance in screening intentions beyond the variables included in the model. The findings suggest that participation in mammography can be driven not only by anxiety or worry but also by positive traits like PC, with meaningful theoretical and practical implications.

Prior work has established PC as a significant predictor of action in emotionally threatening situations (4, 5, 10, 11), but these were typically reactive rather than preventive. By demonstrating that PC predicts engagement in preventive behavior—in this case, mammography screening—our findings suggest that PC also plays a role in motivating behavior aimed at long-term health, even when no immediate situational threat is present. Taken together, these findings suggest that PC may function less as a moderator of emotional processes and more as an independent motivational resource supporting preventive action. Accordingly, PC may represent a meaningful addition to existing models of preventive health behavior.

Our study provides new insights into the relationship between PC and negative emotions. Contrary to the concern that PC might counteract the potential motivational effects of negative emotions, our results did not support this possibility. PC does not appear to diminish the influence of adaptive fear or anxiety; instead, it may complement these emotions and, potentially, compensate when they are less salient.

The finding that PC accounted for screening intentions even when BCW was not a significant predictor suggests that it may serve as an alternative motivational driver for individuals who may be less responsive to fear-based cues. Moreover, PC accounted for screening intentions alongside trait anxiety, suggesting the possibility of distinct yet parallel pathways leading to the same outcome. These findings are consistent with the view that multiple motivational processes can independently contribute to health behavior. From the perspective of Reinforcement Sensitivity Theory (RST; 12), anxiety-related processes are linked to the behavioral inhibition system (BIS), which can motivate vigilance and avoidance in the face of threat. At the same time, they may contribute to adaptive outcomes through improved planning and behavioral regulation (38). PC, as an approach-oriented tendency that supports engagement despite negative emotions, may reflect a distinct motivational pathway functionally related to the behavioral activation system (BAS). Within this framework, PC may also function as a moderator by influencing the extent to which fear-related processes translate into action. However, the lack of a significant interaction provides no evidence that BIS-related and PC-related processes operate synergistically in the present context, and further research is warranted to examine their potential interplay.

The findings of this study have important practical implications. In breast cancer education, incorporating PC into communication strategies may help shift the focus from emotional barriers to personal resources. This approach may be particularly relevant for individuals with low levels of cancer worry, for whom fear-based messages may be less effective. While educational interventions can increase awareness, their impact on behavior remains limited (39). For example, a study conducted in Ghana found that although 73% of students were informed about breast cancer and screening, only 43% reported performing regular self-examination, partly due to low perceived risk (40).

Promoting a *courageous mindset* may help individuals cope with the discomfort associated with mammography and support engagement among those who are hesitant or less responsive to fear-based cues. As proposed by Pajestka and Skałacka (6), an increase in PC could result from learning that being anxious or fearful is not equivalent to being a coward, and that PC manifests despite fear, not because of its absence. However, this proposition was derived as rational, not empirical, and thus should be taken with caution.

In sum, PC may represent a promising target for interventions aimed at increasing engagement in preventive health behaviors, particularly in contexts where fear alone is insufficient to motivate action. Future research could explore whether strengthening PC—for example, through psychological training or educational programs—enhances willingness to participate in screening. Such approaches may complement traditional fear-based interventions by supporting action despite discomfort.

## Limitations

These findings should be interpreted with caution. The cross-sectional design precludes causal inferences, and the observed relationships should be interpreted as associative. Online recruitment via social media may have introduced selection bias, potentially limiting the representativeness of the sample, particularly among older or less digitally engaged women. Self-report measures may also have skewed responses, as reflecting on cancer risk can trigger defensiveness or heightened awareness, and social desirability effects and common method bias cannot be ruled out (41). Additionally, although several relevant covariates were included, potential confounders such as healthcare access or systemic barriers were not accounted for and may have influenced the observed relationships. Future research should address these limitations by examining more diverse samples, employing longitudinal or experimental designs, and incorporating additional contextual variables to further clarify predictors of screening behavior. In particular, directly comparing psychological courage with conceptually related constructs (e.g., self-efficacy or emotion regulation) would help clarify its incremental contribution.

## Data Availability

Publicly available datasets were analyzed in this study. This data can be found here: Open Science Framework (OSF) https://osf.io/vd2p9/?view_only=a0cdcf560f954a498e6df0758e39d348.
