# Supplementary material for: Dare to take care of yourself: the relationship between psychological courage and the intention to undergo mammography screening
Source: Front Public Health. 2026 Jun 9;14:1778940. doi: 10.3389/fpubh.2026.1778940 (PMC13287013; doi:10.3389/fpubh.2026.1778940)
Supplement: Supplementary file 1 [file Data_Sheet_1.docx]

**Supplement to “Dare to Take Care of Yourself: The Relationship Between Psychological Courage and the Intention to Undergo Mammography Screening.”**

| Table 1S | | |
| --- | --- | --- |
| Sociodemographic characteristics of the participants | | |
| Sample structure | n | % |
| 45–49 | 114 | 39 |
| 50–59 | 114 | 39 |
| 60–69 | 55 | 18.8 |
| ≥70 | 9 | 3.1 |
| Education |  |  |
| Primary | 1 | 0.3 |
| Vocational | 15 | 5.1 |
| Secondary | 62 | 21.2 |
| Higher | 214 | 73.3 |
| Place of residence |  |  |
| Village | 97 | 33.2 |
| Small city (<20,000 residents) | 68 | 23.23 |
| Medium city (20,000–99,000 residents) | 46 | 15.8 |
| Big city (100,000–500,000 residents) | 19 | 6.5 |
| Very big city (>500,000 residents) | 62 | 21.2 |
| Family history of breast cancer |  |  |
| yes | 222 | 76 |
| no | 70 | 24 |
| Ever underwent mammography |  |  |
| yes | 196 | 67.1 |
| no | 96 | 32.9 |
| *Note*. Some percentages may not add up to 100 due to rounding. | | |

| Table 2S |  |  |  |  |  |  |
| --- | --- | --- | --- | --- | --- | --- |
| Descriptive statistics of the focal study variables. | | | | | | |
|  | Mean | SD | Min | Max | Skewness | Kurtosis |
| intention to undergo mammography | 12.40 | 3.30 | 3.00 | 15.00 | -1.29 | 0.83 |
| PC | 23.23 | 4.12 | 10.00 | 30.00 | -0.39 | 0.08 |
| BCW | 10.58 | 3.81 | 4.00 | 20.00 | 0.23 | -0.64 |
| perceived risk | 5.52 | 1.41 | 2.00 | 10.00 | -0.10 | 0.88 |
| trait anxiety | 31.18 | 8.69 | 12.00 | 50.00 | 0.13 | -0.55 |
| *Note.* PC – psychological courage, BCW – breast cancer worry. | | | | | | |

**Supplementary Material: Psychological Courage Measure**

The present study assessed psychological courage using the Courage Measure (CM;Norton & Weiss, 2009) in its shortened, six-item version. Psychological courage is conceptualized as persistence or approach behavior despite the presence of fear or anxiety, reflecting an approach-oriented response to threat.

The scale consists of six items rated on a 7-point Likert scale (1 = strongly disagree to 7 = strongly agree). A sample item is: “I tend to face my fears.” Scores are calculated as the mean of all items, with higher scores indicating greater psychological courage.

The CM is grounded in theoretical accounts of courage as an adaptive process enabling individuals to engage with fear-inducing situations, consistent with definitions emphasizing persistence despite emotional distress (Norton & Weiss, 2009; Putman, 1997; Rachman, 2004).

Previous research has demonstrated that the scale exhibits a unidimensional structure, good internal consistency, and satisfactory validity. In particular, prior studies have shown its associations with resilience, risk-taking, and behavioral approach, supporting its role as a distinct psychological construct (Pajestka, 2023; Pajestka & Poraj-Weder, 2024; Pajestka & Skałacka, 2025).
